# Incidental and symptomatic renal tumors: impact on patient survival

**DOI:** 10.1590/S1516-31802002000600002

**Published:** 2002-11-01

**Authors:** Marcos Francisco Dall'Oglio, Miguel Srougi, Pierre Damião Gonçalves, Kátia Leite, Luciano Nesrallah, Flávio Hering

**Keywords:** Carcinoma, Renal, Cell, Diagnosis, Incidental, Survival, Carcinona, Célula, Renal, Diagnóstico, Incidental, Rim

## Abstract

**CONTEXT:**

Renal cell carcinoma is the third most frequent genitourinary neoplasia, and there is currently an increase in the incidental diagnosis of tumors confined to the kidneys.

**OBJECTIVE:**

To study the survival of patients with incidental and symptomatic renal tumors who have undergone nephrectomy.

**DESIGN:**

Retrospective.

**SETTING:**

Hospital Sírio Libanês and Beneficência Portuguesa de São Paulo.

**PARTICIPANTS:**

115 patients with diagnosis of renal cell carcinoma, operated on by the same group of surgeons and evaluated by a single pathologist.

**MAIN MEASUREMENTS:**

Sex, age and diagnosis method, analyzed in two groups, according to the tumor diagnosis: Group 1 with incidental diagnosis and Group 2 with symptomatic tumors. The anatomopathological characteristics and patient survival in both groups were evaluated. A statistical analysis was performed using the Student t, chi-squared, log rank and Kaplan-Meyer tests.

**RESULTS:**

Among the studied patients, 59(51%) had an incidental diagnosis, with 78% diagnosed by ultrasonography, 20% by computerized tomography scan and 2% during surgeries; 56 patients (49%) were symptomatic. Tumor locations were equally distributed between the two kidneys, and the surgery was conservative for 24% of the incidental and 9% of the symptomatic group. In the incidental group only one patient had tumor progression and there was no death, while in the symptomatic group there were 5 progressions and 10 deaths. The 5-year specific cancer-free survival was 100% in the incidental and 80% in the symptomatic group (p = 0.001) while the disease-free rate was 98% in the incidental and 62% in the symptomatic group (p < 0001).

**CONCLUSION:**

Incidental renal tumor diagnosis offers better prognosis, providing longer disease- free survival.

## RESUMO

**CONTEXTO:**

O carcinoma de células renais é a terceira neoplasia geniturinária em frequência, havendo atualmente um aumento no diagnóstico incidental de tumores restritos ao rim.

**OBJETIVO:**

Verificar a sobrevida dos pacientes com tumores de rim incidentais e sintomáticos submetidos a nefrectomia.

**TIPO DE ESTUDO:**

Retrospectivo.

**LOCAL:**

Hospital Sírio Libanês e Beneficência Portuguesa de São Paulo.

**PARTICIPANTES:**

115 pacientes com diagnóstico de carcinoma de células renais e operados por um mesmo grupo de cirurgiões, ava- liados por um único patologista.

**VARIÁVEIS ESTUDADAS:**

Sexo, idade e método diagnóstico, analisados em dois grupos conforme o diagnóstico do tumor: Grupo 1 com diagnóstico incidental e Grupo 2, com tumores sintomáticos. São também avaliadas as características anatomopatológicas da peça operatória e sobrevida dos pacientes nestes dois grupos. Foi realizada análise estatística através do teste t de Student, qui quadrado, log rank test e Kaplan-Meyer.

**RESULTADOS:**

Dos pacientes estudados, 59 (51%) tinham diagnóstico incidental, sendo 78% diagnosticados pelo exame ultra-sonográfico, 20% por tomografia computa- dorizada e 2% em transoperatório; já 56 pacientes (49%) apresentaram-se sintomáticos. Houve um equilíbrio quanto à lateralidade, sendo a cirurgia conservadora em 24% dos incidentais e 9% dos sintomáticos. No grupo incidental, apenas um paciente teve pro-gressão e nenhum óbito, ao passo que os sintomáticos tiveram 5 progressões e 10 óbitos. A sobrevida câncer-específica em cinco anos foi de 100% para incidentais e 80% para os sintomáticos (p = 0,001) e a sobrevida livre de doença foi de 98% para os incidentais e 62% para os sintomáticos (p < 0,001).

**CONCLUSÃO:**

O tumor de rim diagnosticado incidentalmente oferece melhor prognóstico, proporcionando maior sobrevida livre de doença.

## INTRODUCTION

The incidence of renal cell carcinoma (RCC) and the diagnosis of localized tumors are increasing.^1,2^ It was predicted that the worldwide mortality due to RCC would reach 100,000 cases in the year 2000.^3^ The risk of RCC after the age of 40 years is 1.34%, and the risk of death is over 0.5%.^4^

The most common symptoms are hematuria, low back pain and palpable mass, and these events occur in isolation in 35-59%, 34-41% and 30-45% of the cases, respectively.^5-7^ The 5-year survival rate for symptomatic tumors is 30-83% while for incidental tumors it is 83-95%^8-13^ and, according to tumor staging, the rate is 91% for PT1, 74% for PT2, 67% for PT3 and 32% for PT4.^14^ Metastatic disease is seen in 25-40% of the cases at diagnosis,^15,16^ with a 5-year survival rate of 13%.^17^

Twenty years ago, the incidental case rate was less than 5%, but there has been an increase of more than 50% in early diagnosis up to the present day, due to the advent of non-invasive radiological techniques such as ultrasonography and computerized tomography (CT) scans. This has allowed small lesions with favorable prognosis and low incidence of metastasis to be discovered.^8,18,19^

Nonetheless, there are no indications of methods for predicting the behavior of small tumors discovered incidentally.^20^ Many authors question the factors that might influence the behavior of such neoplasia, believing that incidental tumors may display biological behavior that differs from that of symptomatic tumors.^8,21,22^ Would there be a lower degree of malignity and/or slower growth?^23^

Looking for answers to such questions, this study had the objective of analyzing the survival of patients with incidental and symptomatic RCC.

## METHODS

A retrospective non-controlled study was made of 128 patients who underwent renal surgery for RCC between January 1988 and July 1999, with the operation being performed by the same group of surgeons. From this, 115 patients were selected. Their mean age was 59.1 years (range: 9-87) and the median was 60 years, and there were 86 males (74.8%) and 29 females (25.2%).

The complementary diagnostic tests that confirmed the extensive renal lesion were ultrasonography, excretory urography, CT scan, nuclear magnetic resonance and arteriography.

### Criteria for inclusion and exclusion

All patients operated on for RCC who had complete files were included. Thirteen patients were excluded, because of insufficient data and pathological material for eight patients, follow-up of previous RCC in four cases, and von Hippel-Lindau disease in one case.

The follow-up was undertaken from the consultation office. When more than three months had elapsed since the preceding consultation, telephone calls was made to inquire about the patient's current status. In this follow-up, consultations took place every three months during the first year, semiannually from the second to the fifth year and yearly thereafter.

### Evaluation criteria

The reason that led the patient to the doctor was identified and the patients were divided into two groups, according to the diagnosis of the primary tumor.

1. Incidental: patients with findings of extensive renal lesion identified in radiological examinations at routine health checkups or because of complaints unrelated to RCC.
2. Symptomatic: patients with symptoms related to RCC.

The patients were followed up for periods ranging from 2 to 138 months, with a median of 26 months for incidental cases and 33 months for symptomatic cases.

### Statistical analyses

Statistical analyses were performed via the Student t and chi-squared tests. The specific cancer-free survival and disease-free rates were calculated using the Kaplan-Meyer curves and the log rank test was used to compare differences in the survival of the groups. The statistical significance level utilized was p < 0.05.

## RESULTS

In the incidental group there were 59 patients (51%) and in the symptomatic group, 56 (49%). The lesions of the incidental group were identified by ultrasonography in 46 patients (78%), by CT scan in 12 (20%) and one case was identified during the operation (2%).

The male to female ratios were, respectively, 45:14 in the incidental and 41:15 in the symptomatic group. The mean ages of males and females were, respectively, 62.7/ 57.2 in the incidental and 57.2/55.0 in the symptomatic group (non-significant).

In the incidental group, 45 radical (76%) and 14 conservative (24%) surgeries were performed, while in the symptomatic group 51 radical (91%) and 5 conservative (9%) nephrectomies were performed.

The dominant cell type was clear cells in 59% (68), chromophile cells in 23% (26), chromophobe in 10%, and sarcomatous in 8%.

When the tumor size was related to the type of clinical presentation, it was noticed that lesions of between 0.5 and 4 cm were present in 30 (51%) of the incidental and 11 (20%) of the symptomatic cases; between 4.1 and 7 cm in 21 (36%) and 22 (39%); between 7.1 and 10 cm in 5 (8%) and 14 (25%); and tumors greater than 10 cm in 3 (5%) and 9 (16%), respectively (p = 0.001). The mean tumor size was 4.6 ± 2.3 cm (range 0.5 to 13) in the incidental and 7.3 ± 3.6 cm (range 1.5 to 19.5) in the symptomatic group (p = 0.001).

With regard to the pathological staging, among the incidental cases there were 47 patients in stage PT1, 5 in stage PT2 and 7 in stage PT3. Among the symptomatic cases there were 27 patients in stage PT1, 11 in stage PT2, 10 in stage PT3 and 8 in stage PT4 (p < 0.001).

**Figure 1:**
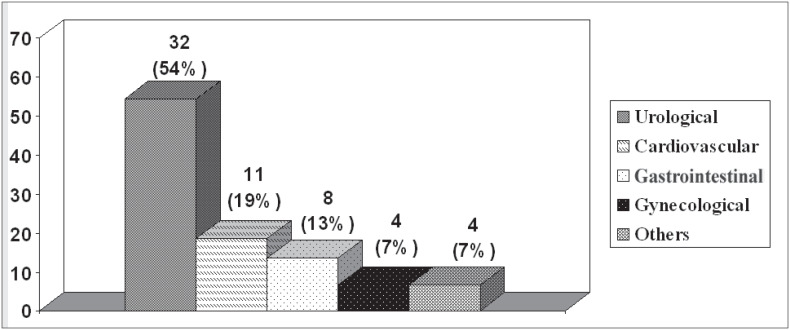
Reasons for consultations sought by patients with incidental diagnosis.

**Figure 2:**
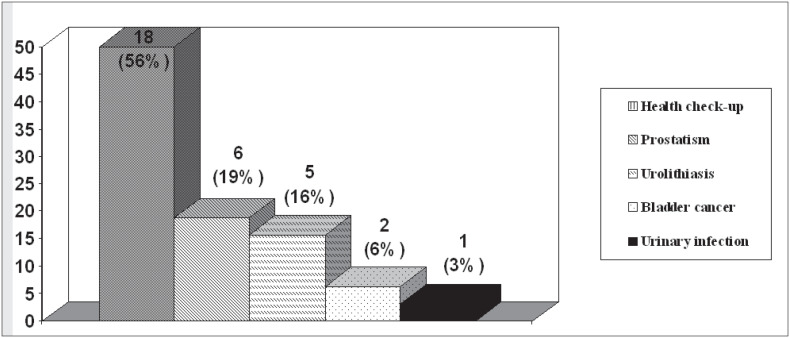
Reasons for urological consultations sought by patients with incidental diagnosis.

**Figure 3:**
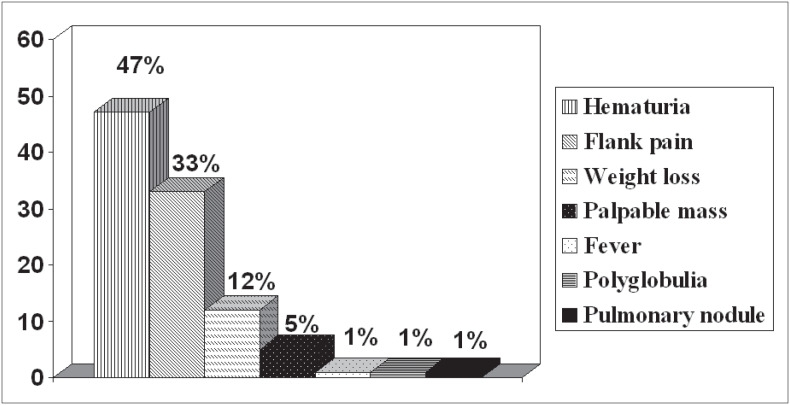
Reasons for consultations sought by patients in the symptomatic group.

The reasons for consultations sought with different specialties are seen in Figure 1, and subsequently the urological consultations were separated out (Figure 2). The main complaints in the symptomatic group are seen in Figure 3. Complaints were balanced with regard to which of the kidneys was involved and the presentation.

By the end of the study, only one patient in the incidental group showed progression of the disease and there was no death, while in the symptomatic group there were five progressions and 10 deaths. Only four patients were lost during the follow-up period of 2 to 138 months (Table).

**Table.** Survival situation at the end of the study, according to the presentation.

Situation at the end of the study	Incidental group	Symptomatic group	Total
Alive, without evidence of disease	54	36	90
Alive, with disease (progression of disease)	1	5	6
Death due to the kidney cancer	0	10	10
Death due to other causes (unrelated to the disease)	4	1	5
Lost from the follow-up	0	4	4
**Total**	**59**	**56**	**115**

The specific cancer-free survival (p = 0.001) and disease-free survival (p < 0.001) are shown in Graphics 1 and 2 respectively.

**Graph 1:**
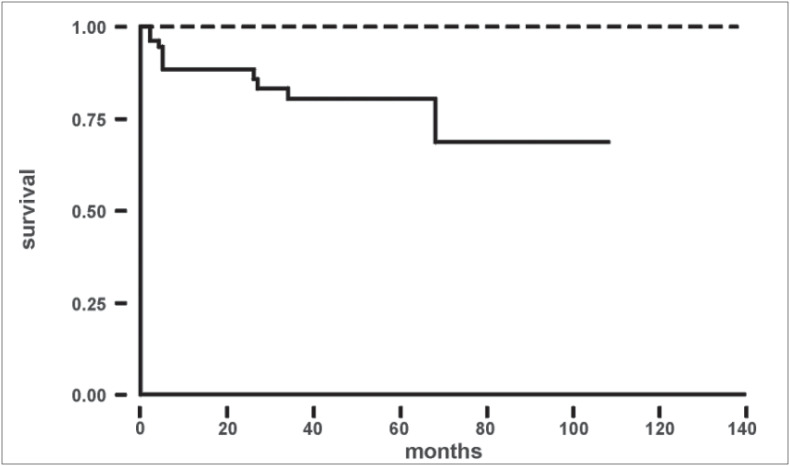
Specific cancer-free survival curve according to presentation. Log rank test*: c^2^_1gl_ = 10.16; p = 0.001; Incidental group = () dotted line; Symptomatic group = (——) full line*

**Graph 2:**
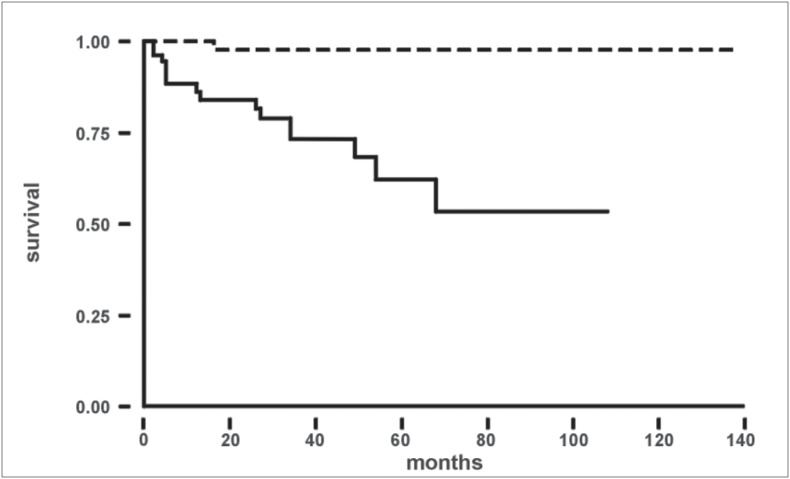
Disease-free survival curve according to presentation. Log rank test*: c^2^_1gl_ = 12.48; p < 0.001; Incidental group = () dotted line; Symptomatic group = (——) full line*

## DISCUSSION

This study shows that incidental tumors have a better prognosis and provided longer disease-free survival then the symptomatic tumors did during the period analyzed. Although this study was retrospective, 96.6% of the patients were followed up and thus the results can be considered reliable.

Several authors have reported high percentages of incidental tumors identified via ultrasonography, with rates of 38%-97%.^24-28^ In this study, 78% of the diagnoses were made by ultrasound and 20% by CT scan. These data show that the ultrasonography examination has an important role in the early detection of such tumors, considering that it is used for evaluating cardiac, hepatic, gallbladder, pancreas and gynecological diseases as well as in routine health check-ups. Currently, two- thirds of incidental tumors are found by other physicians, in non-urological situations.^25,29^ The finding of a 50% incidental diagnosis rate in this study is probably due to the fact that the research was performed in a specialized clinic. In the present study, tumor location was equally distributed between the two kidneys, but some series have shown greater numbers of incidental cases in the right kid- ney.24,26,27

Currently, large centers defend the use of some type of screening.^9^ The screening of high risk populations, especially for those undergoing dialysis or with von Hippel-Lindau disease, and for those over 50 years of age, is advised.^30,31^ On the other hand, some other groups reject this approach in the belief that the cost/benefit relationship of such screening is not well de-fined.^24,25,32^ However, rapid routine examination of the kidneys during ultrasonography of other organs is also defended.^25,33^

Hematuria was the main complaint in the symptomatic cases, in 47% of the cases in this study, and only one patient showed the classical triad (hematuria, back pain and palpable mass) that in the past was seen in 10% of cases^5^ and is now only rarely seen.^30^

In the last few years there has been an increase in the detection of incidental tumors,^18^ directly related to the use of imaging exams, especially ultrasonography and CT scans.^18,34,35^ The "internist's tumor"^36^ can thus be renamed the "radiologist's tumor",^30^ because of the identification of RCC at its early stages, thereby increasing the apparent disease prevalence.^37^ These tumors are frequently small and found in routine examinations and evaluations of other diseases,^38,39,40^ with greater incidence among younger males and females with a useful working life.^25^ Considering that RCC occurs in the proportion of 3:1 for men to women, in the fifth and sixth decades of life, most of these patients might be diagnosed by their own urologist. The practical implication of this study is that incidental diagnosis of renal carcinoma will dramatically change the prognosis for patients with RCC, offering a real possibility of cure for most patients.

In a study by Nakano et al.,^23^ incidental diagnosis was more prevalent among the elderly, which can be related to the lower degree of malignity and slower growth of RCC. In the present series, there was no statistical difference regarding age at diagnosis, for either group.

In the incidental group, conservative surgery was performed in 24% of the cases, while this was done in 9% of the symptomatic cases. Such surgery was performed in tumors smaller than 4 cm, in accordance with advice in the literature,^41,42^ with no recurrence. Obviously, the difference in the use of conservative surgery between the groups is justified by the smaller size of incidental tumors, which forms an indication for preservation of the renal unit.

The 5-year survival rate found in different studies for asymptomatic and symptomatic patients is, respectively, 83-95% and 30-83%.^8-13^ In the present series, by the end of the study period, in the incidental group there was only one case of progression, while among the 56 patients in the symptomatic group, 5 showed progression and 10 died. The probability of disease-free survival after 5 and 9 years was respectively, 98% and 98% for the incidental group and 62% and 53% for the symptomatic group (p < 0.001).

It has been shown that 80% of RCC metastases occur by the third year of follow-up.^43^ With this in mind and with the objective of more clearly showing the survival in time units, a cutoff was established for patients with a follow-up equal to or longer than 30 months. This confirmed the better survival curves for the incidental group (p < 0.001).

## CONCLUSION

RCC that is found incidentally offers a better prognosis for patients because it provides for longer disease-free survival.
